# Transcriptome-Based Comparative Expression Profiling of Sweet Potato during a Compatible Response with Root-Knot Nematode *Meloidogyne incognita* Infection

**DOI:** 10.3390/genes14112074

**Published:** 2023-11-13

**Authors:** Yeon Woo Sung, Jaewook Kim, Jung-Wook Yang, Donghwan Shim, Yun-Hee Kim

**Affiliations:** 1 Department of Biology Education, IALS, Gyeongsang National University, Jinju 52828, Republic of Korea; 2 Division of Applied Life Science (BK21 Plus), Gyeongsang National University, Jinju 52828, Republic of Korea; 3 Department of Biological Sciences, Chungnam National University, Daejeon 34134, Republic of Korea; 4 Department of Crop Cultivation & Environment, Research National Institute of Crop Science, RDA, Suwon 16429, Republic of Korea

**Keywords:** responsive gene, root-knot nematode, susceptible cultivars, sweet potato, transcriptome

## Abstract

*M. incognita*, a root-knot nematode (RKN), infects the roots of several important food crops, including sweet potato (*Ipomoea batatas* Lam.), and severely reduces yields. However, the molecular mechanisms underlying infection remain unclear. Previously, we investigated differential responses to RKN invasion in susceptible and resistant sweet potato cultivars through RNA-seq-based transcriptome analysis. In this study, gene expression similarities and differences were examined in RKN-susceptible sweet potato cultivars during the compatible response to RKN infection. Three susceptible cultivars investigated in previous research were used: Dahomi (DHM), Shinhwangmi (SHM), and Yulmi (YM). Of the three cultivars, YM had the highest number of genes with altered expression in response to infection. YM was also the cultivar with the highest susceptibility to RKN. Comparisons among cultivars identified genes that were regulated in more than one cultivar upon infection. Pairwise comparisons revealed that YM and DHM shared the most regulated genes, whereas YM and SHM shared the lowest number of regulated genes. Five genes were up-regulated, and two were down-regulated, in all three cultivars. Among these, four genes were highly up-regulated in all cultivars: germin-like protein, anthranilate synthase α subunit, isocitrate lyase, and uncharacterized protein. Genes were also identified that were uniquely regulated in each cultivar in response to infection, suggesting that susceptible cultivars respond to infection through shared and cultivar-specific pathways. Our findings expand the understanding of the compatible response to RKN invasion in sweet potato roots and provide useful information for further research on RKN defense mechanisms.

## 1. Introduction

Plant-parasitic nematodes cause an estimated average crop yield loss of 10.7% in major crops and 14% in economically important crops [1]. These losses are estimated to exceed USD 100 billion yearly [1]. Root-knot nematodes (RKN; *Meloidogyne* spp.) are sessile obligate endoparasitic plant-parasitic nematodes that infect a wide variety of plant species and are among the most damaging crop parasites worldwide [2]. RKNs parasitize plant root systems, impacting the absorption of water and nutrients and leading to effects at the whole plant level. Crop yields are then reduced, resulting in significant economic losses [3]. The most economically important RKN species worldwide is *M. incognita*, which has characteristic ‘root-eating’ knots or nodules [4,5]. RKNs cause dramatic morphological and physiological changes in plant cells. Some plant genes are subverted by RKNs to establish feeding cells, and the expression of several plant genes has been confirmed in response to infection [6,7].

Several studies have reported physiological and biochemical changes in a range of plant species during RKN infection [8]. During infection, physical changes become apparent in the root epidermis and activation of early signaling mechanisms also occurs. These processes cause biochemical changes through the production of reactive oxygen species (ROS), and the expression of downstream genes is regulated through hormonal changes. However, the gene expression and signaling processes that occur in individual species during RKN infection are not always consistent among plant species. Changes in gene expression during RKN infection in a range of crop plants, including sweet potato, were recently investigated using transcriptome analysis. The roots of RKN-infected crop plants, including tomato, rice, eggplant, alfalfa, and tobacco, revealed changes in the expression of genes related to the cell wall morphology and development, primary and secondary metabolism, and defense signaling during RKN-compatible responses [9,10,11,12,13,14,15]. Changes to transcription factors and differences in hormone signaling, redox protein, and disease resistance proteins were also identified during compatible and incompatible responses. These physiological changes impacted the susceptibilities of different varieties within the same plant species.

Sweet potato (*I. batatas* L.) is an important crop species that is primarily grown in parts of Asia and Africa. Sweet potato serves as a direct source of human nutrition, providing energy, fiber, and antioxidants (including pigment antioxidants). Sweet potato is also used as an industrial raw material for animal feed, starch, and alcohol [16,17]. The main threats to sweet potato are fungal and viral diseases, but recent climate warming continues to increase the damage caused by plant-parasitic nematodes [1,18,19]. Sweet potato is a particularly well-suited host for *M. incognita*, which causes severe damage to storage roots and occurs primarily in tropical and subtropical regions, including South Korea and Japan [20,21]. Research on RKN resistance in sweet potato has been conducted on the selection and physiological characteristics of RKN-resistant sweet potato cultivars [20,21,22,23,24]. Several studies have been conducted comparing the physiological characteristics and resistance levels of various cultivars based on the reported resistant cultivar Tanzania and the susceptible cultivar Beauregard [25,26]. Additionally, a QTL analysis related to RKN resistance traits was conducted using the resistant cultivar Tanzania and the susceptible cultivar Beauregard [27,28,29]. In another study, RKNs infecting sweet potato were classified by race, and SNPs of each race associated with infectivity were identified [30]. The genome-wide DNA molecular markers for the RKN resistance trait were also analyzed in the resistant sweet potato cultivar J-Red and the susceptible cultivar Choshu according to each RKN race [31,32].

Our previous research used transcriptome analysis to examine sweet potato gene expression during RKN infection in susceptible and resistant cultivars [33]. Further transcriptome analysis also confirmed expression differences in susceptible and resistant cultivars experiencing compatible and incompatible infection responses [34]. The expression levels of several candidate genes thought to be involved in RKN resistance were correlated with susceptibility and resistance in several sweet potato cultivars. Finally, the response to RKN infection in susceptible and resistant cultivars was recently shown to involve ROS regulation mechanisms [35]. The sweet potato cultivars used in the previous studies included cv. Yulmi (YM), Shinhwangmi (SHM), and Dahomi (DHM), which are susceptible to RKN infection and showed a compatible response [34]. Of these, YM exhibited higher sensitivity to RKN infection than SHM and DHM [23,24]. In this study, a transcriptional analysis was used to examine similarities and differences in the compatible response in the three susceptible sweet potato cultivars.

## 2. Materials and Methods

### 2.1. Plant Materials

Three sweet potato (*I. batatas* L. Lam) cultivars were obtained from the Bioenergy Crop Research Center of the National Crop Research Institute (Muan Rural Development Administration, Korea). The cultivars used for transcriptome analysis in the previous study [34] and this study were RKN-susceptible cultivars Dahomei (DHM), Shinhwami (SHM), and Yulmi (YM). Sweet potato plants were inoculated with *M. incognita* according to the method of Lee et al. [34]. Fifteen plants of each variety were planted in perforated 500 cm^3^ clay pots in autoclaved sterile sand:soil mixture (50:50). The composition of the soil mixture consisted of coco peat (20%), peat moss (59.26%), perlite (20%), dolomite (0.632%), moisturizer (0.008%), and fertilizer (0.1%). The pots were grown in a greenhouse maintained at 25–30 °C and, 2 weeks after planting, approximately 3000 *M. incognita* eggs were sown into the soil of each pot and covered with a layer of moist sand. Four weeks after inoculation, roots were collected and egg numbers were visually assessed.

### 2.2. Functional Annotation

Functional annotation of differentially expressed genes (DEGs) in three susceptible sweet potato cultivars from previously studied RNA-seq data (PRJNA429283) was performed via sequence similarity search using the BLAST program against the *Arabidopsis thaliana* protein database with an e-value threshold of 1 × 10^−5^. Gene Ontology (GO) enrichment analyses were performed using DAVID (https://david.ncifcrf.gov/, accessed on 7 September 2022). To perform MapMan analysis, *Arabidopsis* homolog gene IDs and DEG fold changes from the three sweet potato cultivars were mapped to biotic stress pathways (https://mapman.gabipd.org/home, accessed on 7 September 2022). A pictorial representation of the biological stress pathway was downloaded from the MapMan website.

### 2.3. RNA Isolation and Gene Expression Analysis

Total RNA was isolated from each sweet potato root sample using TRIzol reagent (Invitrogen, Carlsbad, CA, USA) and treated extensively with RNase-free DNase I to remove contaminating genomic DNA. Quantitative real-time PCR was performed using a Bio-Rad CFX96 thermal cycler (Bio-Rad, Hercules, CA, USA), with EvaGreen fluorescent dye, according to the manufacturer’s instructions. Linear data were normalized to the average threshold cycle (Ct) of the ADP-ribosylation factor (ARF) reference gene [36]. Gene-specific primers are listed in Supplemental Table S1.

### 2.4. Statistical Analysis

Data were analyzed using one-way analysis of variance (ANOVA). The level of statistical significance was set at *p* < 0.05. Subsequent multiple comparison of means was performed using a least significant difference (LSD) test. All statistical analyses were performed using the Statistical Package for the Social Sciences (SPSS 27, IBM, Armonk, NY, USA).

## 3. Results

### 3.1. Identification of the RKN-Compatible Response in Susceptible Sweet Potato Cultivars

A previous analysis confirmed that three sweet potato cultivars, Dahomi (DHM), Shinhwangmi (SHM), and Yulmi (YM), were susceptible to infection by the RKN *M. incognita* and exhibited compatible responses [34,35]. In addition, our earlier transcriptome analysis of the induced defense response during RKN infection identified putative unique transcripts in pairwise sample comparisons as reliable DEGs (fold change > 2, Kal’s z-test FDR *p* < 0.005). Among the identified DEGs, 116 and 55 were significantly up- and down-regulated during infection, respectively, in the three RKN-susceptible cultivars compared with uninfected controls [34]. The three cultivars exhibited different RKN susceptibilities, with YM being more susceptible to RKN infection than DHM and SHM [34,35].

**Figure 1:**
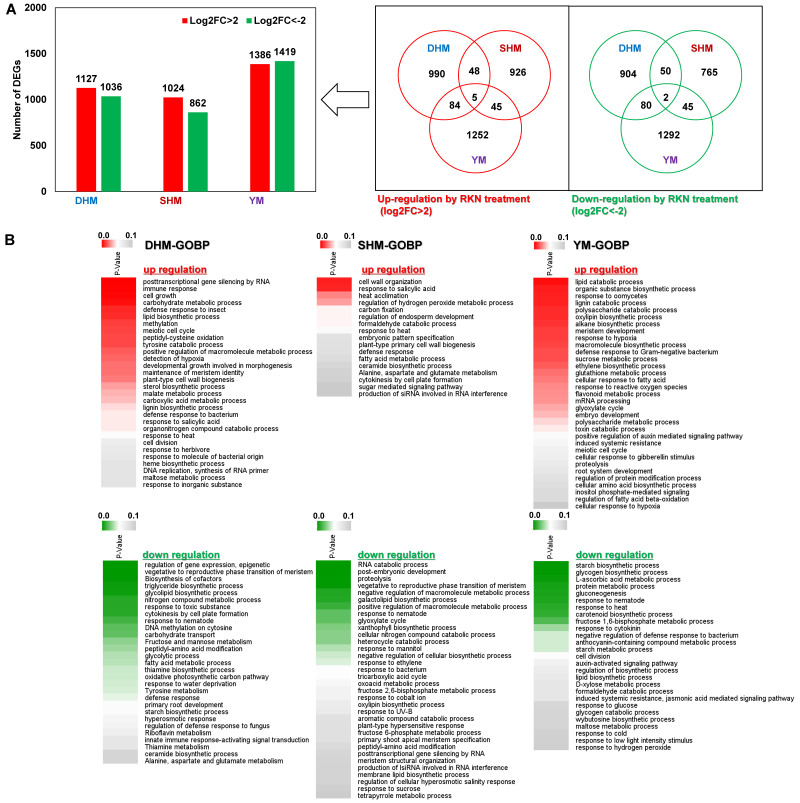
Identification of DEGs in response to RKN infection in three RKN-susceptible sweet potato cultivars. (**A**) Numbers of DEGs up- or down-regulated by Log2FC > 2 (fold change > 4) in response to RKN infection. (**B**) GO biological process category heatmaps of DEGs in control (uninfected) and treated (RKN-infected) conditions for each cultivar. Heatmaps show Benjamini–Hochberg-adjusted (*p* < 0.05) for DEGs enriched for specific GO terms in the biological process (GOBP) category. DHM, Dahomi; SHM, Shinhwangmi; YM, Yulmi.

In this study, to analyze the differences in the compatible response of each RKN-susceptible sweet potato cultivar during *M. incognita* infection, putative unique transcripts as reliable DEGs (fold change > 4 or <−4) were identified for further analysis with RNA-seq (Figure 1A and Supplemental Table S2). One week after RKN infection, 1127, 1024, and 1386 genes in DHM, SHM, and YM, respectively, were up-regulated by Log2FC > 2 (fold change > 4). Similarly, 1036, 862, and 1419 genes were down-regulated in DHM, SHM, and YM, respectively (Figure 1A). The largest numbers of DEGs, both up- and down-regulated, were seen in YM, which was most the susceptible to RKN infection. DEGs were functionally characterized by comparing their predicted encoded gene products with the *A. thaliana* protein database, followed by a GOBP (Gene Ontology Biological Process) enrichment analysis of the annotated genes (Benjamini–Hochberg-adjusted *p* value < 0.05) (Figure 1B). The GOBP term analysis revealed diverse response patterns to RKN infection for the three cultivars. In DHM, the up-regulated major DEGs included post-transcriptional gene silencing by RNA, immune response, and cell growth; the down-regulated major DEGs included regulation of the gene expression epigenetic, vegetative to reproductive phase transition of meristem, and biosynthesis of cofactors. In SHM, the up-regulated major DEGs included cell wall organization, response to salicylic acid (SA), and heat acclimation; the down-regulated major DEGs included the RNA catabolic process, post-embryonic development, and proteolysis. In YM, the up-regulated major DEGs included the lipid catabolic process, organic substance biosynthetic process, and response to oomycetes; the down-regulated major DEGs included the starch biosynthetic process, glycogen biosynthetic process, and L-ascorbic acid metabolic process. Among the up-regulated DEGs, the response to SA was consistent in DHM and SHM, and the meiotic cell cycle was consistent in DHM and YM. Among the down-regulated DEGs, the response to nematode was consistent in all three cultivars, vegetative to reproductive phase transition of meristem and peptidyl-amino acid modification were consistent in DHM and SHM, and the starch biosynthetic process was consistent in DHM and YM. MapMan ontology analysis revealed the genes involved in the response to nematode infection, including genes involved in pathogen recognition, defense response signaling, plant hormones, cell wall metabolism, protein degradation, redox state, transcription factors (TFs), and secondary metabolism (Supplemental Figure S1). In all three RKN-susceptible cultivars, respiratory burst-mediated signaling was up-regulated during the compatible response to RKN infection, whereas hormone signaling pathways, cell wall metabolism, redox proteins, TFs, and secondary metabolic biosynthesis pathways were differently regulated in the three cultivars. In DHM, the phytohormone SA and jasmonic acid (JA) signaling pathways were up-regulated, and WRKY TFs were also up-regulated. However, several glutathione-S transferases (GSTs) and TFs, such as ERF, bZIP, MYB, and DOF, were down-regulated. SHM showed up-regulation of the phytohormone ethylene (ET), SA, brassinosteroid (BR), and abscisic acid (ABA) signaling pathways, as well as up-regulation of several disease resistance (R) genes, pathogenesis-related (PR) proteins, and ERF TFs. However, many genes related to the phytohormone JA and auxin signaling pathways were down-regulated, and genes encoding β-glucanase (GLU), peroxidase (POD), and the TFs bZIP and DOF were also down-regulated. In YM, phytohormone SA and JA signaling-related genes, TF WRKY, and secondary metabolite biosynthesis-related genes were up-regulated. Many R genes; PR proteins; TFs such as ERF and DOF; and phytohormone signaling-related genes such as auxin, BR, and ABA, were down-regulated.

### 3.2. Shared Compatible Responses to RKN Infection in RKN-Susceptible Sweet Potato Cultivars

**Figure 2:**
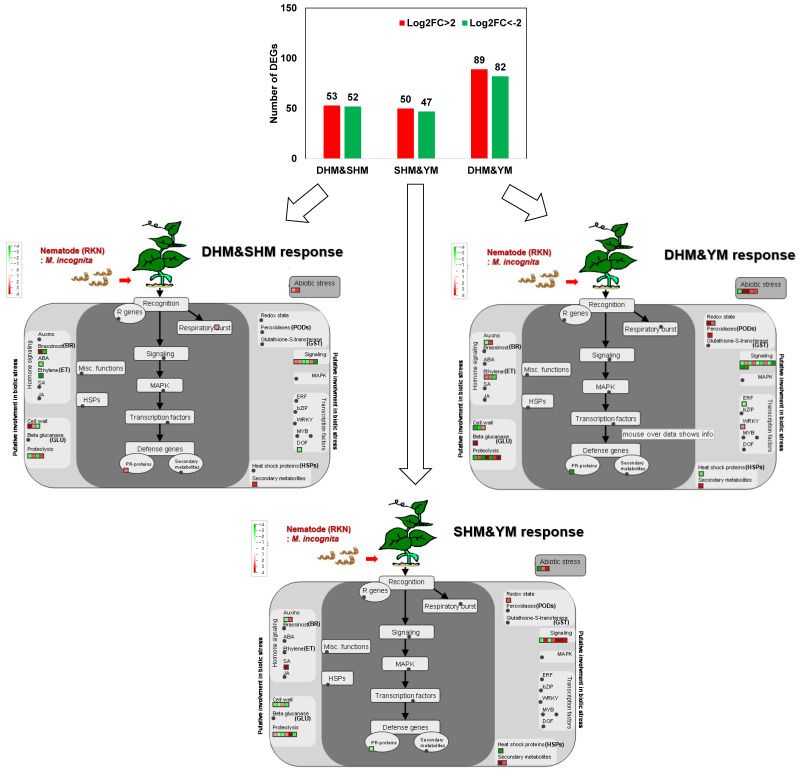
Identification of shared DEGs in pairwise comparisons among three RKN-susceptible sweet potato cultivars. Numbers of DEGs up- or down-regulated by Log2FC > 2 (fold change > 4) in response to RKN infection in pairwise comparisons (**upper**) and MapMan analysis of regulated genes for each cultivar pair (**lower**). An overview of gene expression patterns (log2FC) in infected plants relative to the untreated control is shown. Dots indicate the different paralogous genes encoding proteins related to steps in the defense response. Red dots indicate up-regulation and green dots indicate down-regulation. DHM, Dahomi; SHM, Shinhwangmi; YM, Yulmi.

The three susceptible sweet potato cultivars exhibited similarities and differences in their transcriptional changes during RKN infection (Figure 2). Five genes exhibited increased expression and two genes had down-regulated expression in all three cultivars (Figure 1A). In pairwise comparisons, DHM and SHM shared 53 up-regulated and 52 down-regulated genes, and SHM and YM shared 50 up-regulated and 47 down-regulated genes (Figure 2). The largest number of shared genes was seen with DHM and YM, which shared 89 up-regulated and 82 down-regulated genes with a >4-fold expression change. MapMan analysis was used to identify genes with up-regulated or down-regulated expression of Log2FC > 2 or Log2FC < −2 (Figure 2). Genes that were up-regulated in both DHM and SHM included respiratory burst-related oxidase genes, PR genes, secondary metabolite-related genes, and genes responsive to abiotic stress. Shared down-regulated genes were ABA and ET signaling-related genes, and Dof TF. The genes up-regulated in SHM and YM included redox-related genes, secondary metabolite-related genes, and genes involved in phytohormone SA signaling, multiple signal transduction, and abiotic stress responsiveness. The down-regulated genes included cell wall metabolism-related genes, PR genes, and heat shock proteins (HSPs). The genes up-regulated in DHM and YM included redox-related genes and GLU, POD, WRKY TF, and secondary metabolite-related genes. The down-regulated genes included ERF TF, PR protein, HSP, and several cell wall metabolism and signaling genes.

**Figure 3:**
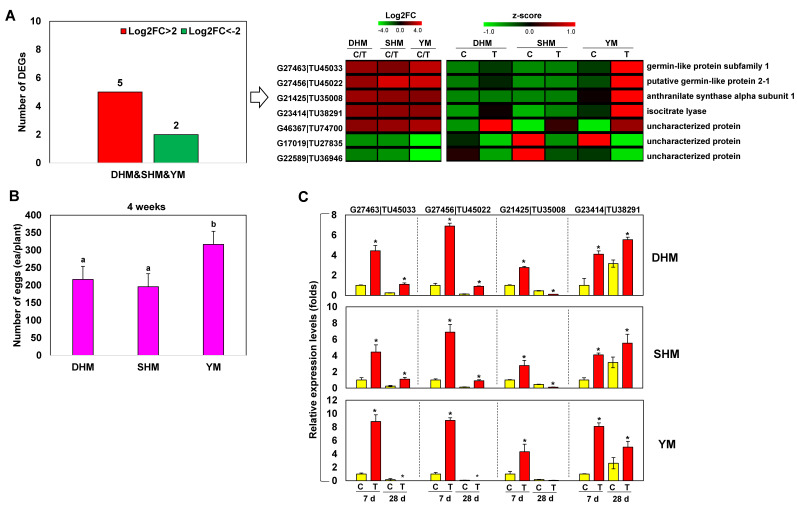
Expression profiling of shared DEGs in pairwise comparisons among three RKN-susceptible sweet potato cultivars. (**A**) DEGs up- or down-regulated by Log2FC > 2 (fold change > 4) in response to RKN infection in all three cultivars. (**B**) Numbers of egg masses in sweet potato roots 4 weeks after RKN treatment. (**C**) Expression profiling of shared compatible response-related marker genes. Relative transcript levels of DEGs encoding germin-like protein subfamily 1 member 20 (G27463|TU45033), putative germin-like protein 2-1 (G27456|TU45022), anthranilate synthase α subunit 1 (G21425|TU35008), and isocitrate lyase (G23414|TU38291) are shown. DHM, Dahomi; SHM, Shinhwangmi; YM, Yulmi; C, control; T, treatment. Bars denoted with the same letter are not significantly different. Bars denoted with the asterisk indicates significantly different compared with control (*p* = 0.05) according to Dunnett’s test.

Finally, five genes were up-regulated and two genes were down-regulated by >4-fold in all three cultivars (Figure 3A). The five genes with increased expression were G27463|TU45033 (germin-like protein subfamily 1 member 20), G27456|TU45022 (putative germin-like protein 2-1), G21425|TU35008 (anthranilate synthase α subunit 1), G23414|TU38291 (isocitrate lyase), and G46367|TU74700 (uncharacterized protein). The two down-regulated genes (G17019|TU27835 and G22589|TU36946) were uncharacterized proteins. Consistent with previous studies [34,35], differences in egg number were seen for the three cultivars 4 weeks after RKN infection, with the highest egg numbers observed in YM (DHM: 216.67 ± 3.2, SHM: 195.67 ± 10.3 and YM: 316.67 ± 3.1) (Figure 3B). The expression patterns of four of the commonly up-regulated genes, G27463|TU45033, G27456|TU45022, G21425|TU35008, and G23414|TU38291, which were up-regulated 1 and 4 weeks after RKN infection, were examined using qRT-PCR (Figure 3C). In all three cultivars, expression of three of the four genes increased during the first 7 days after infection and had decreased by 28 days post-infection. However, expression of G23414|TU38291 (isocitrate lyase) increased until 28 days post-infection in DHM and SHM, but showed decreased expression at 28 days in YM.

### 3.3. Cultivar-Specific Compatible Responses to RKN Infection in RKN-Susceptible Sweet Potato Cultivars

**Figure 4:**
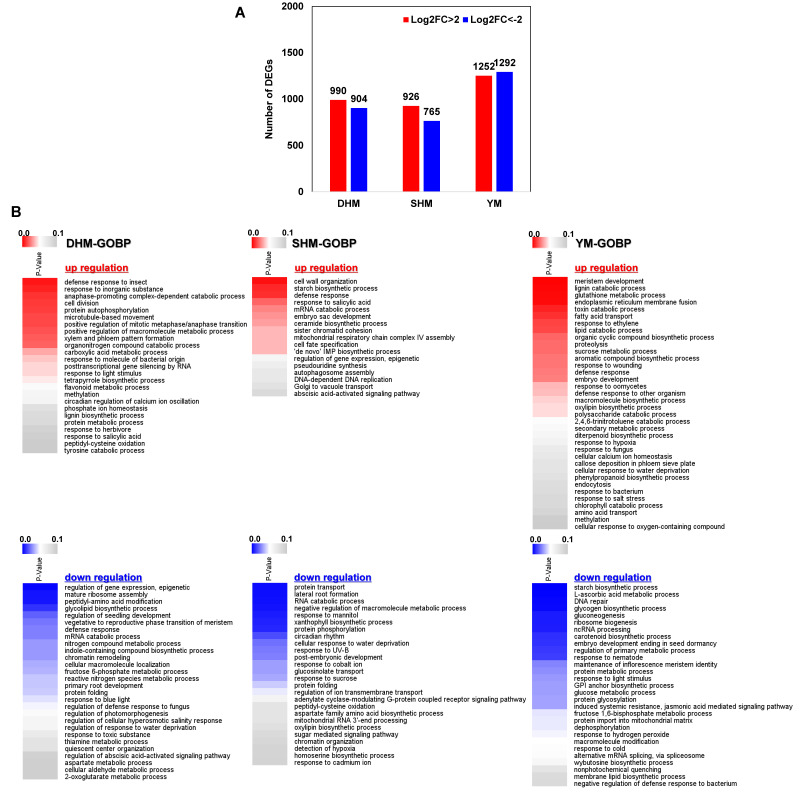
Identification of cultivar-specific DEGs in response to RKN infection. (**A**) Numbers of DEGs uniquely up- or down-regulated by Log2FC > 2 (fold change > 4) in response to RKN infection in each cultivar. (**B**) GO biological process category heatmaps of DEGs in control (uninfected) and treated (RKN-infected) conditions for each cultivar. DHM, Dahomi; SHM, Shinhwangmi; YM, Yulmi.

The three RKN-susceptible sweet potato cultivars used in this study, DHM, SHM, and YM, exhibited different susceptibilities to RKN infection. To investigate these differences, cultivar-specific DEGs with a >4-fold up- or down-regulation upon infection were identified (Figure 4A). There were 990 DHM-specific up-regulated genes and 904 down-regulated genes, 926 SHM-specific up-regulated genes and 765 down-regulated genes, and 1252 YM-specific up-regulated and 1292 down-regulated genes. GOBP term analysis was used to assess the functions of specifically enriched genes for each cultivar (Figure 4B). In DHM, the up-regulated major DEGs included defense response to insect, response to inorganic substance, and anaphase-promoting complex-dependent catabolic process. The down-regulated major DEGs in DHM included regulation of gene expression epigenetic, mature ribosome assembly, and peptidyl-amino acid modification. In SHM, the up-regulated major DEGs included cell wall organization, starch biosynthetic process, and defense response. The down-regulated major DEGs in SHM included protein transport, lateral root formation, and RNA catabolic process. In YM, the up-regulated major DEGs included meristem development, lignin catabolic process, and glutathione metabolic process. The down-regulated major DEGs in YM included starch biosynthetic process, L-ascorbic acid metabolic process, and DNA repair. Among the up-regulated DEGs of each cultivar, response to SA was present in the DEGs of DHM and SHM, and methylation was found in the DEGs of DHM and YM. In the down-regulated DEGs, protein folding was found in both DHM and SHM. Expression changes and predicted gene functional analysis were confirmed using MapMan analysis (Supplemental Figure S2). DHM-specific up-regulated genes included genes related to phytohormone SA and JA signaling and various secondary metabolism-related genes. DHM-specific down-regulated genes included phytohormone signaling (BR, ABA, and ET), TFs (ERF and MYB), and redox-related genes (POD, GST, and HSP). SHM-specific up-regulated genes included genes related to BR, ET, and SA signaling, and the TFs ERF and MYB. R genes, PR proteins, and several proteolysis-related genes were also specifically up-regulated in SHM. The SHM-specific down-regulated genes included auxin and JA signaling genes, WRKY TFs, and redox-related genes (POD and HSP). The YM-specific up-regulated genes included genes involved in SA and JA signaling and respiratory burst-related signaling, as well as POD, GST, and WRKY TFs. Several genes related to cell wall metabolism and secondary metabolites were also up-regulated in YM. The YM-specific down-regulated genes included auxin, BR, and ABA signaling genes, as well as GLU.

**Figure 5:**
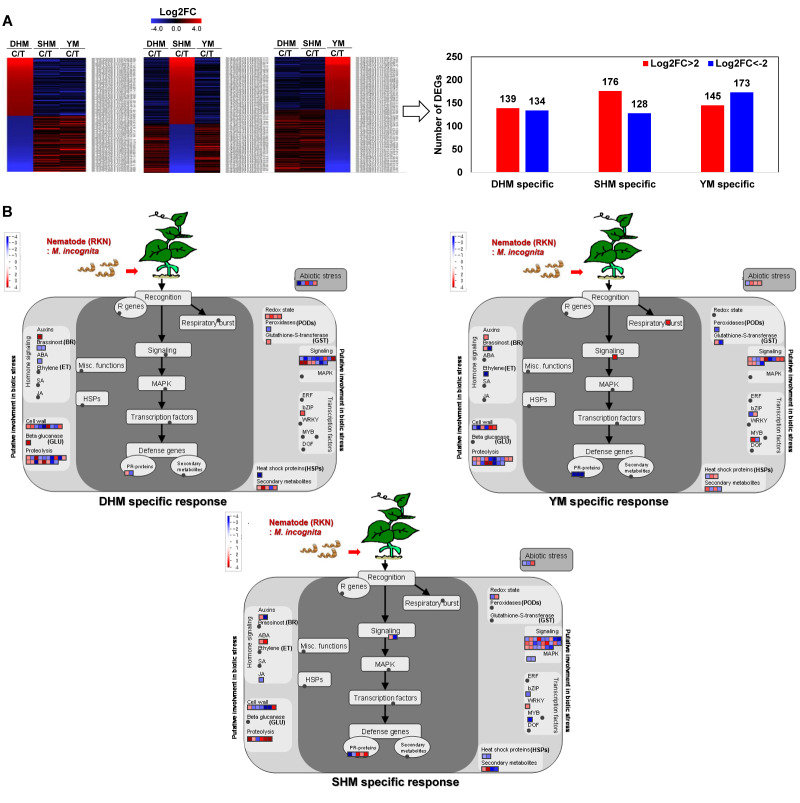
Refinement of cultivar-specific DEGs in response to RKN infection. (**A**) DEGs with >4-fold increased expression in one cultivar and no increase or reduced expression in the other two cultivars, or vice versa for down-regulated genes. (**B**) MapMan analysis of refined cultivar-specific compatible responses to RKN infection. An overview of gene expression patterns (log2FC) in infected plants relative to the untreated control is shown. Dots indicate the different paralogous genes encoding proteins related to steps in the defense response. Red dots indicate up-regulation and blue dots indicate down-regulation. DHM, Dahomi; SHM, Shinhwangmi; YM, Yulmi.

To further refine the genes with strong cultivar-specific expression responses, genes were selected that displayed a >4-fold increased expression in one cultivar and no increase or reduced expression in the other two cultivars, or vice versa for down-regulated genes (Figure 5A and Supplemental Table S3). Using this classification, 139 genes were specifically up-regulated in DHM and 134 genes were down-regulated. In SHM, 176 genes were specifically up-regulated and 128 genes were down-regulated. In YM, 145 genes were specifically up-regulated and 173 genes were specifically down-regulated. A subsequent MapMan analysis identified cultivar-specific RKN-responsive functional genes (Figure 5B). In DHM, genes involved in auxin signaling, GLU, redox reactions, GST, and bZIP TFs were specifically up-regulated, while BR and ABA signaling, POD, and HSP genes were specifically down-regulated. In SHM, ABA signaling-related genes, WRKY TFs, and several proteolysis-related genes were specifically up-regulated, while JA signaling, MAPK, bZIP, and MYB TFs, and HSP genes were specifically down-regulated. Finally, in YM, genes related to respiratory burst, auxin signaling, and abiotic stress response genes were up-regulated and PR protein and POD were specifically down-regulated.

**Figure 6:**
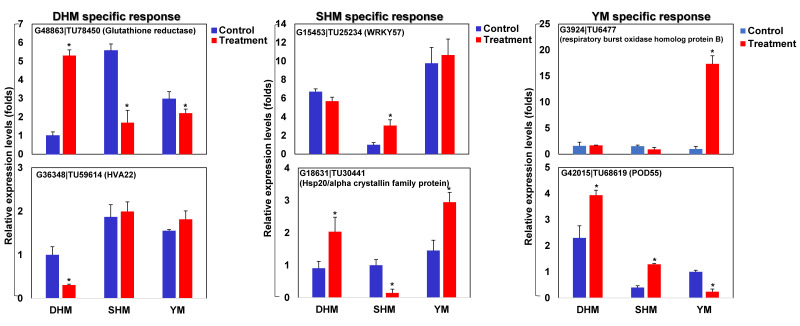
Expression profiling of refined cultivar-specific compatible response-related marker genes. Relative transcript levels of DEGs encoding glutathione reductase (G48863|TU78450), HVA22 (G36348|TU59614), WRKY57 (G15453|TU25234), Hsp20/α crystallin family protein (G18631|TU30441), respiratory burst oxidase homolog protein B (G3924|TU6477), and POD55 (G42015|TU68619) are shown. DHM, Dahomi; SHM, Shinhwangmi; YM, Yulmi; C, control; T, treatment. Bars denoted with the asterisk indicates significantly different compared with control (*p* = 0.05) according to Dunnett’s test.

Selected cultivar-specific genes were assessed using qRT-PCR analysis (Figure 6). G48863|TU78450 (glutathione reductase) showed a specific increase in response to RKN infection only in DHM, with decreased expression seen in SHM and YM. The expression of G36348|TU59614 (HVA22) was reduced only in DHM, with no change of responses in SHM or YM. G15453|TU25234 (WRKY57) increased specifically in SHM, with no response changes in DHM or YM. G18631|TU30441 (Hsp20/α crystallin family protein) expression decreased in SHM and increased in DHM and YM. G3924|TU6477 (respiratory burst oxidase homolog protein B) showed an increased expression in YM, with no change in response in DHM and SHM. G42015|TU68619 (POD55) exhibited decreased expression only in YM, with increased expression in DHM and SHM.

## 4. Discussion

The compatible response between the RKN *M. incognita* and susceptible host plants involves several genes and proteins that mediate plant–nematode interactions [37]. RKNs have developed several strategies to invade and parasitize plants, including using secretions to facilitate the invasion of plant roots. The secretions include cell wall degrading enzymes, effectors, and proteins involved in mimicking host proteins; these promote nematode establishment within plants [5,38]. In the compatible response, RKN can use secretions to regulate the expression of plant genes to favor RKN invasion [39]. This also induces the expression of genes important for plant root establishment and suppresses the expression of defense genes to avoid host plant resistance responses [39,40]. The compatible response exhibited by the host plant in response to RKN infection thus involves the expression and regulation of a wide range of plant genes.

Plant responses during RKN infection were previously examined in several important crop species using RNA-seq transcriptome analysis. Xing et al. [10] performed root transcriptome analysis of resistant (Yuyan12) and susceptible (Changbohuang) tobacco cultivars infected with RKN, with compatible and incompatible responses, respectively. Under normal (uninfected) conditions, 289 DEGs were identified between the two cultivars. When uninfected roots were compared with RKN-infected roots, 2623 and 545 DEGs were identified in Yuyan12 and Changbohuang, respectively. Among these DEGs, genes encoding cell wall modifying proteins, auxin-related proteins, ROS scavenging systems, and TFs involved in various biological and physicochemical processes were significantly expressed in both the RKN resistant and susceptible tobacco cultivars. Zhou et al. [14] examined gene expression changes during the compatible response to RKN invasion in rice roots, and found 952 and 647 genes were differentially expressed 6 days (invasion phase) and 18 days (development phase) after inoculation, respectively. The DEGs were classified into various metabolic and stress response categories, and phytohormones, TFs, redox signaling, and defense response pathways were enhanced during RKN infection. Further analysis using qRT-PCR confirmed that CBL-interacting protein kinase (CIPK) genes (CIPK5, 8, 9, 11, 14, 23, 24, and 31), BR-related genes (OsBAK1, OsBRI1, D2, and D11) and ET signaling-related genes ERF and ERS exhibited expression changes upon RKN infection. A transcriptome analysis of two RKN-susceptible eggplant cultivars also demonstrated differential expression during the compatible response [15], with 5360 DEGs identified in response to RKN infection. GO term analysis showed that these DEGs were mainly involved in response to stimuli, protein phosphorylation, hormone signaling, and plant–pathogen interaction pathway processes. TFs, including MYB, WRKY, and NAC, and various phytohormone-related genes, including ABA and BR, were differentially expressed in the two RKN-susceptible cultivars during RKN infection. Shukla et al. [11] examined expression changes in both RKN-susceptible and RKN-resistant tomato cultivars during RKN infection to investigate compatible and incompatible response mechanisms within the plant and nematode. During the compatible response to RKN infection, 1827 DEGs were identified in the susceptible tomato cultivar alongside 462 DEGs in the RKN. During the incompatible response, 25 DEGs were identified in the resistant tomato cultivar alongside 160 DEGs in the RKN. The tomato genes involved in cell wall structure, development, primary and secondary metabolites, and defense signaling pathways, and the RKN genes involved in host parasitism, development, and defense were identified in the compatible response. In the incompatible response, the tomato genes involved in secondary metabolites and hormone-mediated defense responses were identified, along with RKN genes involved in starvation stress-induced cell death. Thus, compatible and incompatible responses to RKN infection are indicative of RKN susceptibility and resistance, respectively, in a range of crop species. Genes that were differentially expressed during infection among species included those related to cell wall-related proteins, plant hormone signaling, ROS regulation, TFs, and secondary metabolites, all of which are thought to have important roles during the infection process. Our previous study, which used transcriptome analysis to examine responses to RKN infection in susceptible and resistant sweet potato cultivars, revealed the differential expression of several genes during the incompatible and compatible responses [33,34]. Consistent with other studies, cell wall-related proteins, plant hormone signaling, ROS regulation, TFs, and secondary metabolites were putatively identified as playing important roles during RKN infection in sweet potato.

The RKN-susceptible sweet potato cultivars used in our earlier study had different susceptibilities to RKN infection [34,35]. Zhang et al. [15] identified differences in gene expression during infection between two RKN-susceptible eggplant cultivars. This suggests that RKN-susceptible cultivars that show compatible responses to RKN infection each respond to RKN infection through shared and distinct response mechanisms. In this study, DEGs that were up- or down-regulated by >4-fold were selected to investigate compatible response mechanisms seen during RKN infection in three RKN-susceptible sweet potato cultivars (Figure 1). Genes with shared transcription profiles in the three cultivars included those encoding germin-like protein, anthranilate synthase, and isocitrate lyase (Figure 3). Germin-like proteins (GLPs) are plant glycoproteins found in diverse land plants [41]. Several studies have shown that GLPs are stable under heat, extreme pH, and detergent treatment [42], and they are reported to have various oxidase activities, such as superoxide dismutase (SOD), oxalate oxidase (OXO), ADP glucose pyrophosphatase/phosphodiesterase, and polyphenol oxidase activities [43,44,45,46]. From the perspective of plant–microbe interactions, GLPs are considered part of the PR16 protein family [47]. Many GLPs are located in the cell wall and function as cofactors for cell wall strengthening by promoting cross-linking of plant cell wall components. This activity of GLPs increases resistance to infection and involves generating H_2_O_2_ through SOD or OXO activity [48,49]. GLP-induced H_2_O_2_ can initiate the SA and/or JA signaling pathways, leading to the synthesis of PR proteins and the stimulation of plant defenses, respectively [50]. GLPs are also important components of plant host resistance and can be up-regulated and/or activated by a pathogen infection or the application of disease resistance-related chemicals such as H_2_O_2_, SA, and ET [51,52,53]. Anthranilate synthase (AS) catalyzes the first reaction branching from the aromatic amino acid pathway to tryptophan biosynthesis in plants, fungi, and bacteria [54]. While tryptophan is primarily required for protein synthesis in bacteria and fungi, the tryptophan pathway in plants also provides precursors for the synthesis of key secondary metabolites such as major endogenous auxins and indole-3-acetic acid, among others. These metabolites can help protect plants from pathogens and herbivores. AS is a branch point enzyme in aromatic amino acid biosynthesis, so the regulation of AS is important for regulating the flow of intermediates in the synthetic pathway [54]. AS enzyme activity is generally regulated via tryptophan feedback in plants, fungi, and bacteria. Isocitrate lyase (ICL) acts within the glyoxylate cycle to catalyze the breakdown of isocitrate into succinic acid and glyoxylate [55,56]. Together with malate synthase, ICL bypasses the two decarboxylation steps of the tricarboxylic acid cycle and is used in plants, bacteria, and fungi [57]. ICL has also been shown to be important in plant pathogenesis [57]. For several crops, including cereals, cucumbers, and melons, increased expression of genes encoding ICLs was found to increase fungal virulence. For example, increased expression of *ICL* was observed in the fungus *Leptosphaeria maculans* during an infection of canola. Inactivation of the *ICL* gene reduced the pathogenicity of *L. maculans*, likely as a consequence of the fungus being unable to utilize the plant as a carbon source. Therefore, during RKN infection of the three susceptible sweet potato cultivars, GLPs may be associated with a signaling mechanism through ROS regulation, AS may activate the signaling mechanism of infected plant roots through changes in auxin metabolism, and ICL may be linked to the increased RKN pathogenicity. The GLP, AS, and ICL genes, which exhibit similar responses during the compatible response in three RKN-susceptible sweet potato cultivars, may serve as common markers for susceptibility and should be investigated as RKN infection detection markers.

Genes were identified in the three sweet potato cultivars that exhibited cultivar-specific transcriptional changes during infection (Figure 5). Phytohormone signaling components, such as auxin, BR, and ABA, and β-glucanase, redox-related, GST, POD, bZIP TF, and HSP genes were regulated only in DHM during RKN infection. Phytohormone signaling components including ABA and JA; MAPK, HSP, and proteolysis-related genes; and TFs, such as WRKY, bZIP, and MYB were regulated only in SHM. Respiratory burst, auxin signaling, abiotic stress response, PR protein, and POD genes were regulated only in YM. Therefore, as well as the shared responses during infection, the three RKN-susceptible sweet potato cultivars exhibited differences in their compatibility responses due to expression differences in hormonal signaling, ROS-related genes, and TF genes during RKN infection.

## 5. Conclusions

In conclusion, this study provides in depth novel insights into the molecular response mechanisms involved in plant–RKN interactions. The genes involved in compatible responses during RKN infection were identified in three sweet potato cultivars with different susceptibilities to infection by the RKN *M. incognita.* Signal regulation-related genes, including phytohormone signaling, ROS regulation, and TF genes, were identified as candidates for inducing changes in common and/or differential compatible responses during RKN infection in sweet potato. The extensive repertoire of genes identified in this study will greatly facilitate basic and applied research on plant–RKN interactions. For further research, it is believed that a functional analysis of the crucial genes identified through transcriptome analysis will be necessary, and for this purpose, the cut–dip–budding (CDB) delivery system transformation research method will be used as a good technology [58]. In addition, it is believed that additional research will need to further confirm biochemical indicators of genes related to hormone regulation.

## Data Availability

All of the raw data for our analysis was deposited in PRJNA429283.

## Supplementary Materials

The following supporting information can be downloaded at: https://www.mdpi.com/article/10.3390/genes14112074/s1, Figure S1: MapMan analysis of the compatible response to *M. incognita* infection in sweetpotato cultivars; Figure S2: MapMan analysis of cultivar-specific compatible responses to *M. incognita* infection in sweetpotato cultivars; Table S1: Oligonucleotide primers used for qRT-PCR analysis; Table S2: Expression profiles of seleted RKN response genes in the RKN-susceptible three sweetpotato cultivars during infected and uninfected conditions as determined by mRNA seq.; Table S3: Expression profiles of cultivar specific response genes in the RKN-susceptible three sweetpotato cultivars during infected and uninfected conditions as determined by mRNA seq.
