# Supplementary material for: Transcriptome-Based Comparative Expression Profiling of Sweet Potato during a Compatible Response with Root-Knot Nematode Meloidogyne incognita Infection
Source: Genes (Basel). 2023 Nov 13;14(11):2074. doi: 10.3390/genes14112074 (PMC10671793; doi:10.3390/genes14112074)
Supplement: Supplementary file 1 [file genes-14-02074-s001.zip › Sung et al. (2023)-Supplemental Figures-Genes-Revision-1-2023.11.08.pptx]

## Slide 1
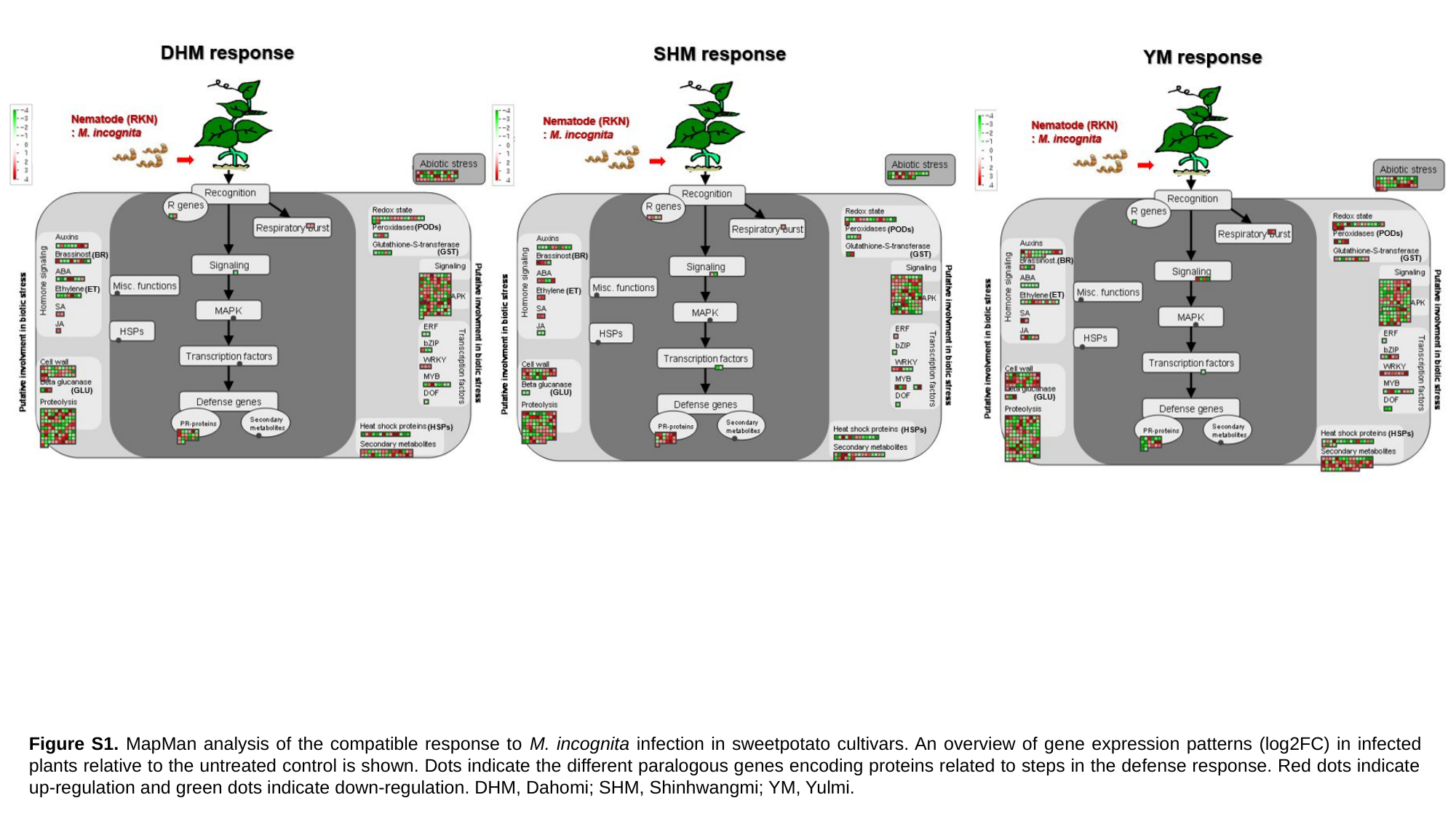

Figure S1. MapMan analysis of the compatible response to M. incognita infection in sweetpotato cultivars. An overview of gene expression patterns (log2FC) in infected plants relative to the untreated control is shown. Dots indicate the different paralogous genes encoding proteins related to steps in the defense response. Red dots indicate up-regulation and green dots indicate down-regulation. DHM, Dahomi; SHM, Shinhwangmi; YM, Yulmi.

## Slide 2
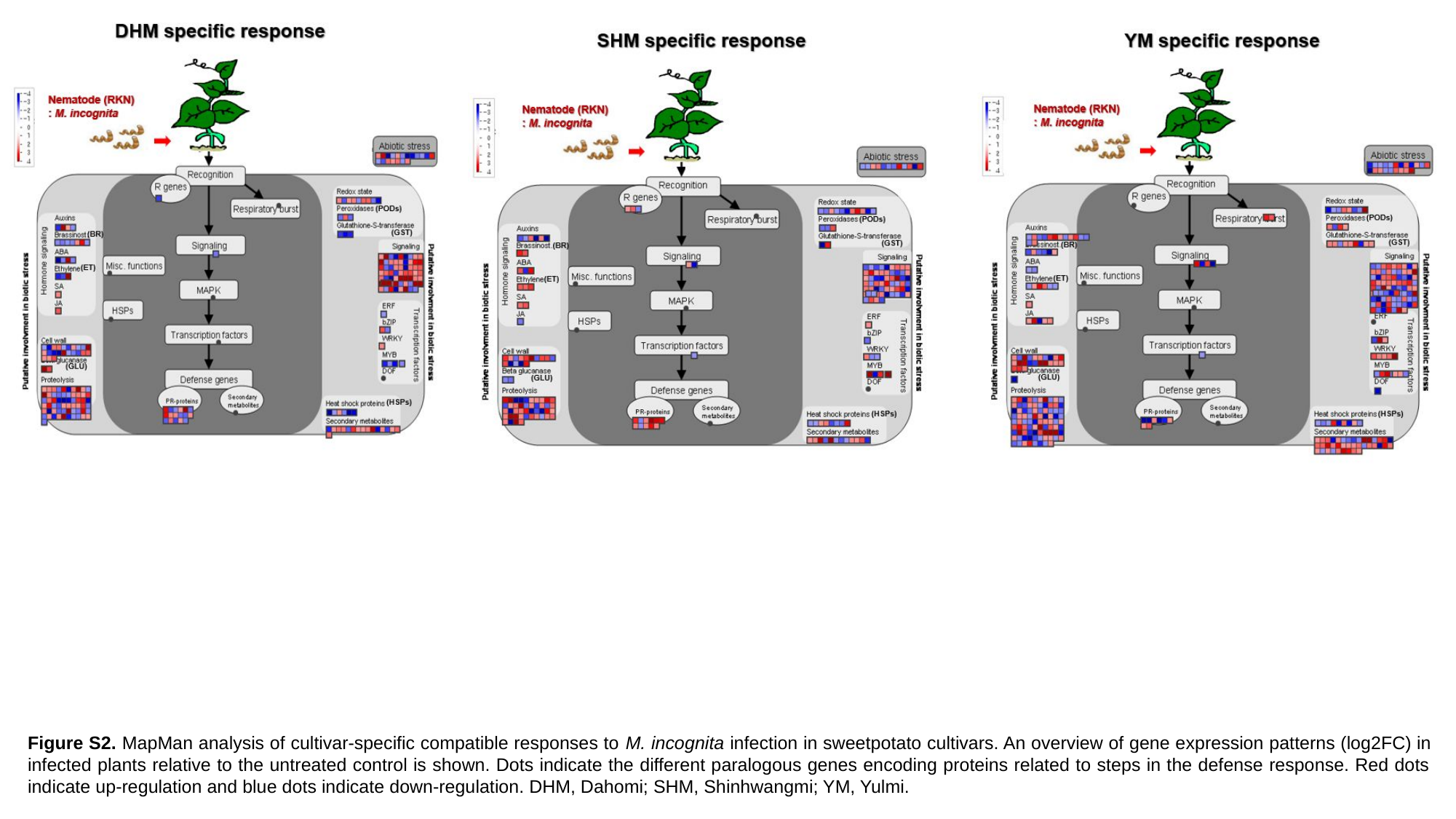

Figure S2. MapMan analysis of cultivar-specific compatible responses to M. incognita infection in sweetpotato cultivars. An overview of gene expression patterns (log2FC) in infected plants relative to the untreated control is shown. Dots indicate the different paralogous genes encoding proteins related to steps in the defense response. Red dots indicate up-regulation and blue dots indicate down-regulation. DHM, Dahomi; SHM, Shinhwangmi; YM, Yulmi.
